# Harnessing synergistic two‐step adsorption of silica/calcium chloride (CaCl_2_) hybrid composites for ammonium removal from aquarium water

**DOI:** 10.1002/wer.70080

**Published:** 2025-06-01

**Authors:** Sarah Farrukh, Xianfeng Fan, Syed Shujaat Karim, Zhibin Yu, Humais Roafi

**Affiliations:** ^1^ Institute for Materials and Processes, School of Engineering The University of Edinburgh Edinburgh Scotland UK; ^2^ Department of Chemical Engineering, School of Chemical and Materials Engineering (SCME) National University of Sciences and Technology (NUST) Islamabad Pakistan; ^3^ Department of Mechanical and Aerospace Engineering, School of Engineering The University of Liverpool Liverpool UK

**Keywords:** adsorption, adsorption kinetics, ammonium ion removal, economical composite adsorbent, water treatment

## Abstract

Ammonium ions, prevalent water contaminants, can become hazardous at high concentrations. To mitigate this risk, various adsorbents have been developed, yet the kinetics of their adsorption processes remain under investigation. In this study, we present the fabrication and comprehensive analysis of pure silica and silica/calcium chloride (CaCl_2_) composites. These materials were characterized using advanced techniques such as scanning electron microscopy (SEM), Fourier‐transform infrared spectroscopy (FT‐IR), X‐ray diffraction (XRD), thermogravimetric analysis (TGA), and differential thermal analysis (DTA). Additionally, pore and salt ratio analyses were conducted using BET and DFT methods. Ammonium ion adsorption efficiency was evaluated through ultraviolet–visible spectroscopy (UV/Vis). The results show that with an increase in the concentration of CaCl_2_, more adsorption was observed. However, the kinetic analysis revealed that the experimental results followed the pseudo‐second‐order model and values of k_2_ show that mass transfer was slow with a 1% CaCl_2_‐based silica composite as compared to other samples. The intramolecular model predicted that the adsorption of ammonium ions was a two‐step process, and the Elovich model fits reasonably, highlighting the presence of physicochemical interactions between ammonium ions and CaCl_2_‐silica composites. Furthermore, these composites proved highly effective in treating aquarium water. The treatment process increased the pH from 6.19 to 6.74 and successfully removed up to 43% of ammonium ions from the aquarium water. These findings highlight the potential of silica/CaCl_2_ composites as a viable adsorbent for ammonium removal for aquarium water purification, offering a significant advancement in maintaining a safe environment for aquatic life.

**Practitioner Points:**

- State‐of‐the‐art characterization techniques confirmed successful fabrication of Silica/Calcium Chloride (CaCl_2_) Hybrid Composites.
- The fabricated composites showed better ammonium ion adsorption, improving the effectiveness of water purification.
- Kinetic studies revealed a pseudo‐second‐order model, with a slower mass transfer rate in low CaCl_2_ concentration composites.
- The intramolecular model indicates a two‐step adsorption mechanism, while the Elovich model validates physicochemical interactions.
- Optimal composite improves the aquarium water pH from 6.19 to 7.08 by the removal ammonium ions.

## Graphical abstract

Combatting Ammonium in Aquarium Waters: Unlocking the Potential of Silica‐CaCl_2_ composites for Safe Water Solutions.

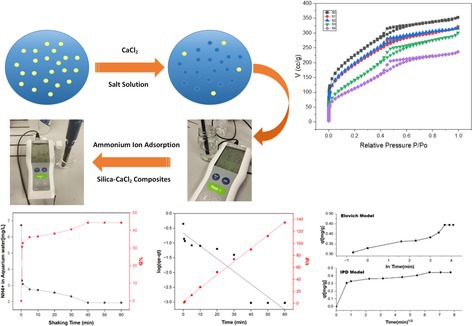

## INTRODUCTION

Ammonia, a natural component of nitrogen cycling, becomes a pollutant when introduced excessively through industrial processes, agricultural runoff, and domestic wastewater discharge (Wang et al., 2023; Wang & Guo, 2020). In wastewater, ammonia predominantly exists as ammonium ions (NH_4_^+^) under lower pH conditions (Alwael et al., 2024). These ions, while essential as a nitrogen source for plant growth and ecosystem health (Adam et al., 2019), can cause severe environmental impacts like water pollution, acidification, and eutrophication when present in excess (Wang & Liu, 2021). Eutrophication, driven by nutrient overloading, disrupts aquatic ecosystems by promoting excessive algae and vegetation growth, degrading water quality and biodiversity (Hwang, 2020). Furthermore, temperature variations amplify the environmental impacts of ammonium pollution, with deviations from the optimal range of 25–32°C harming fish populations by reducing growth and increasing mortality rates (Hwang, 2020). In aquaculture, waste deposits cause pH fluctuations and changes in nitrate levels; however, it is imperative for aquatic plant health and ecosystem balance to keep nitrate concentrations in the range of 0.2–10 mg/l (Zhang et al., 2023). Ammonia, a byproduct of fish farming, disrupts oxygen dynamics and becomes toxic above 0.2 mg/l, while maintaining ammonium levels below 0.10 mg/l at a pH of 6.5–8.5 is critical for aquatic life (Devi et al., 2017; Rosales‐Conrado & Peña‐Martínez, 2023).

To address these challenges, it is imperative to minimize ammonium release into the aquatic environment. Many countries enforce strict regulations, such as China's discharge limit of 5 mg/l for Class I and 8 mg/l for Class II wastewater (Su et al., 2024; Zare et al., 2016), highlighting the critical need for effective ammonium removal strategies. Therefore, various approaches have been developed for ammonium removal from water, each with distinct advantages and limitations. Biological methods, such as nitrification–denitrification, utilize microorganisms to convert ammonium into nitrogen gas or nitrates, commonly applied in wastewater treatment plants (Wang et al., 2012; Zhou, Li, et al., 2024). Chemical methods include precipitation and ion exchange (Lahav & Green, 1998; Lobanov & Poilov, 2006). Among physical methods, adsorption and membrane filtration are notable for their cost‐effectiveness and versatility. The membrane filtration methods, such as reverse osmosis and nanofiltration, separate ammonium based on size and charge differences (Li et al., 2024; Rohani et al., 2021). Whereas, in the adsorption technique, the materials, such as activated carbon and zeolites, offer high surface area and well‐defined porous structures, which help in the binding of ammonium ions as per requirement (Zare et al., 2016). However, these conventional adsorbents often face limitations in adsorption capacity and kinetics, especially under aquaculture conditions.

Over the last several years, a growing number of researchers have focused their efforts on investigating different adsorbents tailored for ammonium sorption, such as (Ismadji et al., 2016) fabricated a bentonite hydrochar‐based composite with a microporous structure, achieving significantly higher adsorption capacity (23.67 mg/g) than pristine bentonite (12.37 mg/g) and biochar (9.49 mg/g), attributed to ion exchange and van der Waals interactions. Similarly, (Widiastuti et al., 2011) and (Kuokkanen et al., 2016) studied natural zeolites and clay‐based adsorbents, demonstrating their non‐toxic and abundant nature, whereas they are restricted by their limited adsorption capacity. Moreover, chemical modifications, such as those applied to NaCl‐treated zeolites (Lin et al., 2013), NaOH‐modified mordenite (Alshameri et al., 2014; Soetardji et al., 2015), and NaOH‐treated clinoptilolite (Wang et al., 2007), significantly enhanced ammonium removal efficiencies up to 99% and 81%, respectively, but raised concerns about the environmental impact of spent adsorbents.

In previous years, to enhance the performance of biochar for ammonium ion adsorption, (Zhang et al., 2020) and (Chen et al., 2017) proposed modifications with bentonite or montmorillonite, while (Gong et al., 2017) demonstrated that Mg‐modified biochar achieved 32 mg/g ammonium adsorption via cation exchange. However, issues such as saturation and frequent regeneration hinder its practical application. Later, composite coagulants like aluminium sulfate and polydimethyldiallylammonium chloride (PDMDAAC), studied by (Adebayo et al., 2021), significantly enhanced coagulation efficiency and ammonia nitrogen removal. However, the presence of residual chemicals limits their use in sensitive environments, such as aquariums. In addition to these studies, ammonium ion‐halide salt interactions have also been explored. (Sharonov & Aristov, 2005) evaluated halide‐based adsorbents (MgCl_2_, CaCl_2_, and BaCl_2_) for ammonia removal, with MgCl_2_ performing best under low temperatures, making it ideal for fish aquariums. (Huang et al., 2021) examined ammonium salt release from weathered crust elution‐deposited rare earth ore and found montmorillonite, among various clays, have the highest adsorption capacity due to its structure, followed by the distinct structure of halloysite, illite, and kaolinite. (Tabares et al., 2001) developed a porous coordination polymer from CuX_2_ salts and 2‐hydroxypyrimidine (Hpymo) for selective ammonium adsorption. (Grekova et al., 2014) studied BaCl_2_ and BaBr_2_ in vermiculite pores, observing consistent sorption enthalpy and varying entropy with increasing ammonia uptake.

Considering the available facts and literature, it is evident that while various adsorbents, such as zeolites, biochars, and modified clays, have been extensively studied for ammonium removal, the potential of ionic halide‐based composites, such as calcium chloride (CaCl_2_), remains largely underexplored, particularly in small‐scale environments like fish aquariums. This study bridges this gap by utilizing silica for its mesoporous structure and CaCl_2_ for its ionic interactions, resulting in a composite with enhanced porosity and improved adsorption capacity. By characterizing these materials utilizing various techniques like SEM, XRD, FT‐IR, TGA/DSC, and BET analysis, and testing their performance in mimicked and aquarium water using UV/VIS spectroscopy, this work explores its suitability for aquaculture. Kinetic analysis further provided insights into the adsorption mechanisms, revealing the potential of silica/CaCl_2_ composites as better and more sustainable solutions for ammonium ion removal.

## MATERIALS AND METHODS

### Materials

The silica was purchased from Sigma‐Aldrich. Calcium chloride (CaCl_2_) salt was purchased from Sigma Aldrich. Nessler's reagent was purchased from Sigma Aldrich, UK. The solvents, acetone and distilled water, were purchased from Sigma Aldrich, UK. The aquarium water used in this experiment was taken from a 10 gal glass fish tank (dimensions: 20x10x12 inches), which had a pH value of 6.19. The filter paper used in the experiment was an MF‐Millipore™ membrane filter, 0.22 μm pore size, 47 mm diameter, made of mixed cellulose esters (MCE) membrane, hydrophilic, purchased from Merck Millipore, U.S.

### Methods

All sample bottles and Petri dishes were thoroughly cleaned using acetone. The silica powder was ground using a mortar and pestle and then dried at 60°C for 24 hours in an oven. To prepare the solution, the optimized quantities of CaCl_2_ salts were dissolved in distilled water and continuously stirred for 2 hours. Subsequently, the ground silica particles were added to the salt solution and left to stir for 24 hours.

The mixture was then filtered using a vacuum filtration setup, and the composite material was collected on filter paper. The residue was heated for 12 hours at a temperature ranging from 60°C to 80°C and collected in sample bottles. This process was repeated for various salt percentages, ranging from 0% to 4%, using the same method as described above. The samples from pure silica to 4 wt.% salt are represented as S0, S1, S2, S3, and S4.

The salt percentages from 0 wt.% to 4 wt.% were selected based on preliminary studies indicating that increasing the CaCl₂ concentration beyond 4 wt.% led to pore blockage in the composite, reducing its adsorption efficiency. To maintain the structural integrity of the composite and optimize adsorption performance, the CaCl₂ content was limited to this range. Further details regarding the rationale behind the selection of this range can be found in the Supplementary Information (Figure S1).

## CHARACTERIZATION

### Scanning Electron microscopy (SEM) of pure and composite silica

The surface morphology and dimensional analysis of pure and composite silica were carried out by Carl Zeiss SIGMA HD VP FEG scanning electron microscopy (SEM) analytical low vacuum SEM, which was provided by JEOL Ltd., Japan. The analysis was performed at two different accelerating voltages, namely 15 kV and 20 kV. To prepare the samples for analysis, a small pinch of the respective silica samples was carefully placed on double‐sided adhesive tape. The opposite side of the tape was securely affixed to sample stubs. Subsequently, a coating of gold was applied to the samples to enhance conductivity and image quality. The prepared samples were then carefully positioned within the SEM chamber for analysis.

### X‐ray diffraction (XRD) analysis

The crystallization analysis of silica and silica composites was carried out using the X‐ray Bruker D‐2 Phaser X‐ray diffractometer, which is equipped with a copper sample holder and utilizes Cu‐Kα radiation with a wavelength of 1.5406 Å. The instrument was operated at a voltage of 30 kV and a current of 10 mA to perform the X‐ray diffraction (XRD) analysis. To prepare the samples for analysis, a small quantity of each pristine and silica composite sample was carefully placed on a sample stub. Subsequently, a glass cover was placed over the samples to ensure uniform distribution and minimal interference during the analysis process.

The prepared samples, securely mounted on the sample stub, were then placed inside the X‐ray diffractometer chamber. The X‐ray beam was directed towards the samples, and the resulting diffraction patterns were recorded. Peaks in the diffraction patterns were analyzed and interpreted to gain insights into the crystalline structure and characteristics of both the pure and silica composite materials.

### Differential scanning calorimetry (DSC)/thermal gravimetric analysis (TGA)

The thermal behavior analysis of the samples was conducted utilizing a Differential Scanning calorimetry (DSC) and thermal gravimetric analysis (TGA) machine, the Netzsch STA 449 F1 Jupiter. To prepare the samples for thermal analysis, the mass of each sample was precisely measured, and each sample was carefully placed in an aluminum pan specifically designed for this purpose. Subsequently, an aluminum lid was securely placed over each sample to encapsulate it within the pan. Prior to analyzing the samples, background peaks were measured to establish a reference baseline. The samples, enclosed within their respective aluminum pans with lids, were then positioned inside the analysis chamber of the DSC and TGA machine. The temperature for the analysis was ramped from 4°C to 400°C, with an increment of 10°C per minute.

### Fourier transform infrared spectroscopy (FT‐IR)

The qualitative analysis of the samples was carried out using an FT‐IR spectrometer (Perkin‐Elmer Frontier). To prepare the samples for FT‐IR analysis, pallets of pure silica and silica composites were prepared and mounted securely in the FT‐IR chamber, ensuring consistent and reliable measurements. The FT‐IR analysis was conducted over a wide range of wave numbers, spanning from 400 cm^−1^ to 4000 cm^−1^, with a high resolution of 4 cm^−1^. This broad range and fine resolution allowed for detailed characterization of the molecular vibrations and functional groups present in the samples.

### Pore structure and salt content analysis

The analysis of specific area and pore volume was conducted using the Quanta chrome Autosorb IQ gas adsorption analyzer. In this physisorption equipment, the BET surface area, pore volume, and pore size distribution were determined. Fabricated samples (pure powder, impregnated powder) were degassed at 110°C for 12 hours before mounting in analyzer. The surface area was calculated using the Brunauer–Emmett–Teller (BET) equation and pore volume was calculated using the DFT method (NLDFT equilibrium model). The analysis of data was carried out by Quantachrome® ASiQwin software. The salt contents in porous samples were calculated using Equation (1).

(1) $${\text{CaCl}}_{2\;\text{content}}=\frac{V_{\text{Pmatrix}}-V_{\text{composite}}}{V_{\text{Pmatrix}}}$$

In this equation, *V_Pmatrix_* represents the pore volume of the pure matrix and *V_composite_* denotes the pore volume of the composite material.

### Ammonium adsorption analysis

The analysis of ammonium adsorption was conducted utilizing the UV/Vis technique. The stock solution for ammonium ions was prepared in accordance with the methodology outlined by (Haseena et al., 2016). Specifically, 3.8190 g of dried ammonium chloride (NH_4_Cl) were dissolved in 1 L of deionized water and stored in a round‐bottom flask.

To establish the calibration curve for ammonium ions, the stock solution was diluted to concentrations of 2 ppm, 4 ppm, 6 ppm, and 8 ppm. The measurements were taken at a wavelength of 400 nm. Since UV spectrophotometers cannot directly detect ammonium ions, Nessler's reagent was introduced into the stock solution at a ratio of 2 ml per 50 ml of stock solution.

The Nessler's reagent changes the color in the sample to a pale yellow or brown, depending on the concentration of ammonium ions present. The concentration of an unknown sample has been calculated using the following Equation (2):

(2) $$Q=\left(\frac{\left(C_{0}-C_{e}\right)V}{m}\right)\times 100$$

Where, *Q* represents the amount of adsorbate, specifically ammonium ions, adsorbed by the adsorbent, expressed as a percentage (%), *C*_*0*_ refers to the initial concentration of ammonium ions in the solution (mg/L), while *C*_*e*_ is the equilibrium concentration of ammonium ions (mg/L). The parameter *V* denotes the volume of the liquid in liters (L), and *m* is the mass of the adsorbent in grams (g) (Trazzi et al., 2024). The calibration curve is represented in Figure S2. Furthermore, to ensure the accuracy of the results, three samples of each concentration were tested to estimate the standard deviation and to eliminate random errors in the analysis. This replication of measurements enhances the reliability of the adsorption data by accounting for experimental variability.

## RESULTS AND DISCUSSION

### Scanning Electron microscopy (SEM)

**FIGURE 1:**
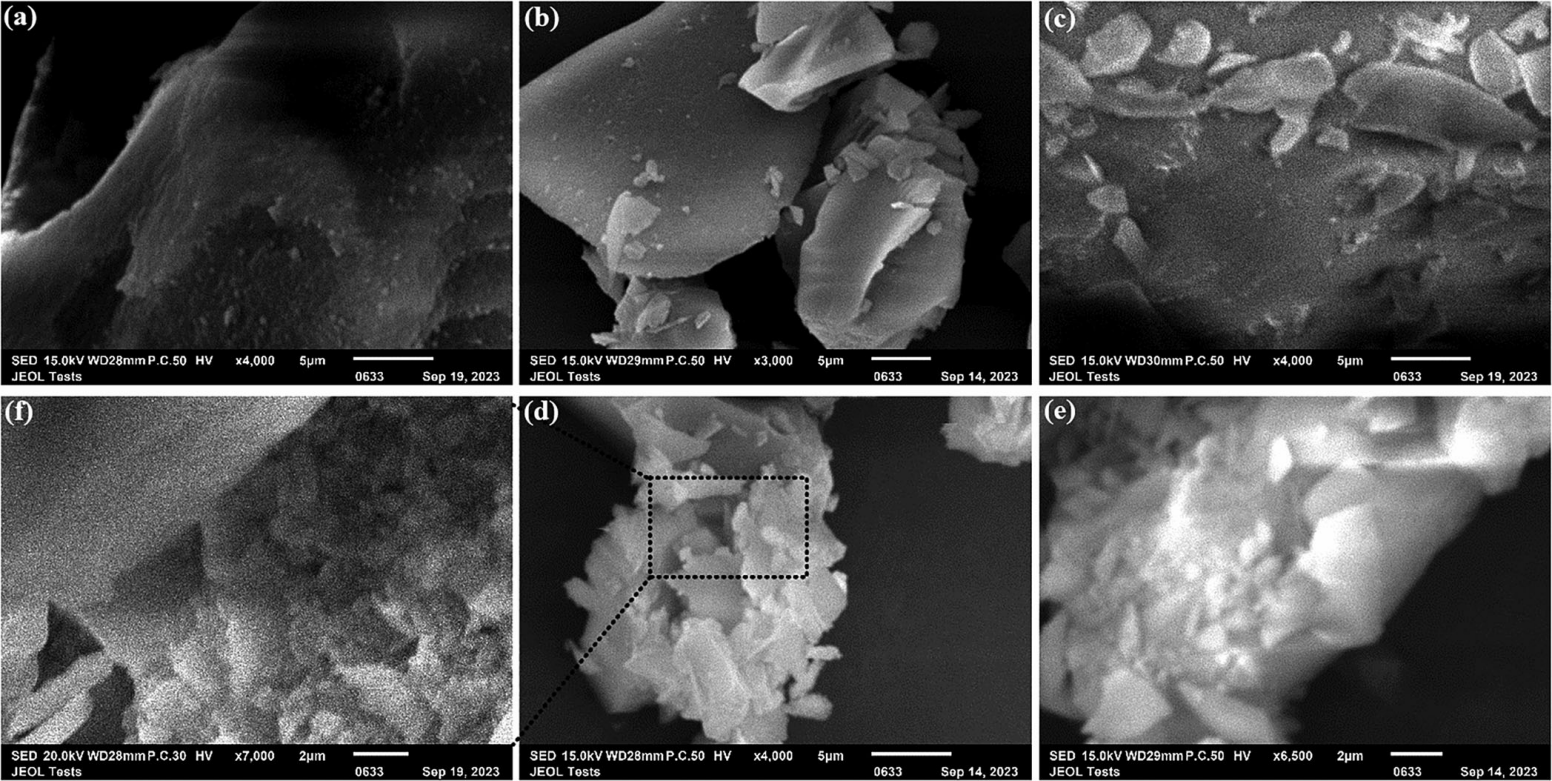
(a) SEM images of pure silica, (b) silica/CaCl_2_–1%, (c) silica/CaCl_2_–2%, (d) silica/CaCl_2_–3%, (e) silica/CaCl_2_–4%, and (f) cavities of silica/CaCl_2_–3%.

The surface morphology of pure silica (S0) and composite silica/CaCl_2_ samples is illustrated in Figure 1a–f. The SEM analysis shows that pure silica (S0) exhibits an uneven surface without the presence of salt flakes, even at ×10,000 magnification. In contrast, as CaCl_2_ is added, starting from sample S1 (1% CaCl_2_), salt flakes become visible on the surface. This trend intensifies with increasing CaCl_2_ content, as evident in sample S4 (4% CaCl_2_), which displays a greater density of salt flakes. These observations confirm the correlation between CaCl_2_ concentration and the presence of salt flakes on the surface of the composite material. At higher magnifications, CaCl_2_ was found to adhere within the cavities and porosity of the silica structure. At higher magnifications, the SEM analysis also revealed that CaCl_2_ salt particles were not only distributed on the surface but also penetrated into the porous structure of the silica. These salt particles tended to adhere within the cavities and smaller pores of the silica matrix, suggesting that the porosity of the silica facilitated the entrapment and integration of CaCl_2_ within the composite. This behavior likely enhances the composite's overall structural integrity and confirms the effective incorporation of CaCl_2_ into the silica framework (Wang et al., 2016).

### Differential scanning calorimetry (DSC) and thermal gravimetric analysis (TGA)

**FIGURE 2:**
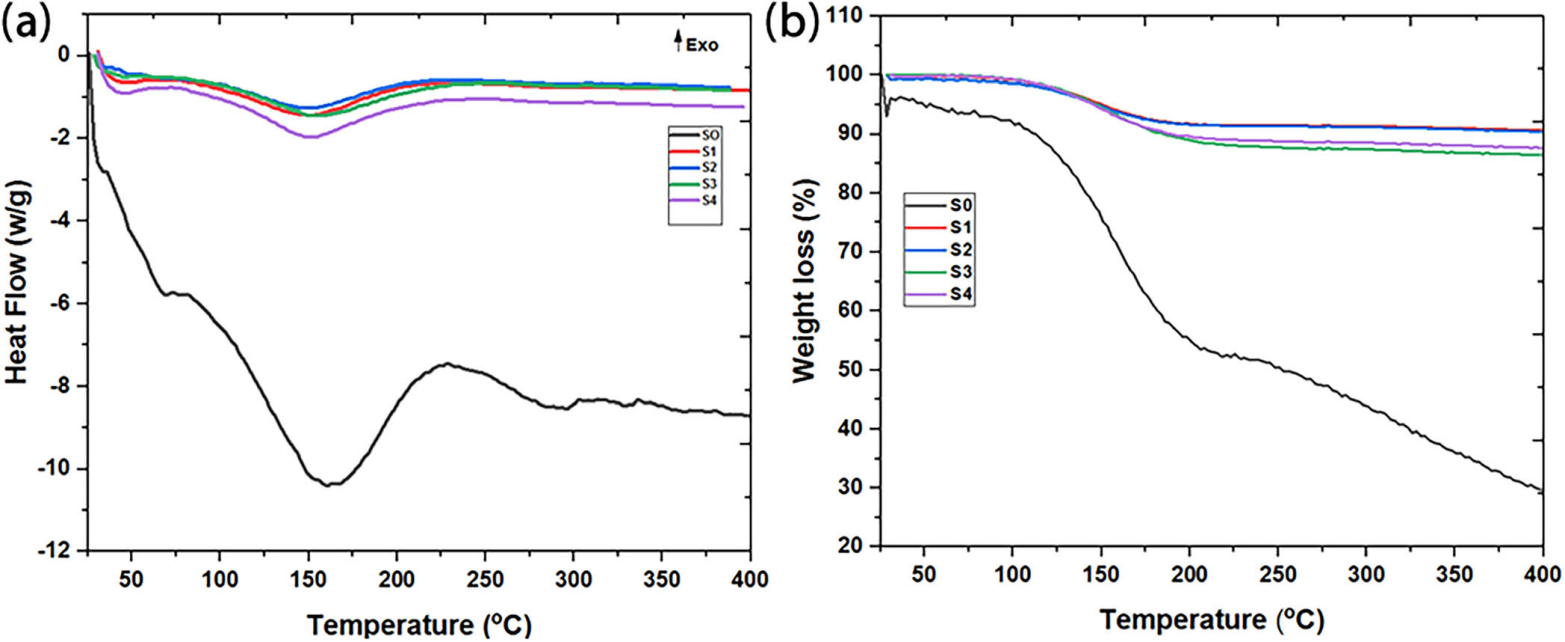
(a) DSC curve, (b) TGA curve.

The differential scanning calorimetry (DSC) and thermal gravimetric analysis (TGA) were employed to evaluate the thermal stability of the silica/CaCl_2_ composites, which is critical for their long‐term use in aquarium water treatment. As illustrated in Figure 2b, the TGA curves reveal a significant weight loss between 70°C and 200°C, corresponding to water loss from the composite material. This weight loss, approximately 9% to 10%, is consistent across the composite samples and is attributed to intramolecular dehydration within the silica/CaCl_2_ matrix (Valor et al., 2002). These findings, supported by previous research conducted by (Li, Lv, et al., 2017; Ma et al., 2021), ensure that the composite maintains its structural integrity under typical aquarium temperature ranges, which usually remain between 22°C and 28°C for most tropical fish species (Yanar et al., 2019).

The DSC curves correspond closely with the TGA results, showing endothermic peaks at the same temperature ranges, indicating the material's absorption of heat during the dehydration process (Zhou et al., 2023). This thermal behavior is important for ensuring that the material remains stable and functional in the aquarium environment, even under slight fluctuations in temperature caused by heaters, lights, or environmental factors. This stability is particularly essential for fish health, as ammonia toxicity increases with temperature, making reliable ammonium ion adsorption a critical factor for maintaining water quality (Randall & Tsui, 2002).

In contrast, the TGA curve for pure silica exhibited a two‐step weight loss pattern. The first step, occurring between 20°C and 100°C, resulted in a minor weight loss of approximately 2% to 3%, attributable to the presence of physically adsorbed water. Subsequently, a more significant weight loss of 58% was observed, which can be attributed to the decomposition of silanol groups as shown in Figure 2a. The large endothermic peak with a ∆H value of −1282 J/g, reflecting the DSC curve, is highlighting the major weight loss. These findings indicate that the blending of silica gel with CaCl_2_ effectively mitigates weight loss at elevated temperatures, ultimately enhancing the thermal stability of the composite. This ensures that the material can withstand temperature variations without significant degradation, thereby maintaining its effectiveness and longevity in removing ammonium ions from aquarium water.

### X‐ray diffraction (XRD) analysis

**FIGURE 3:**
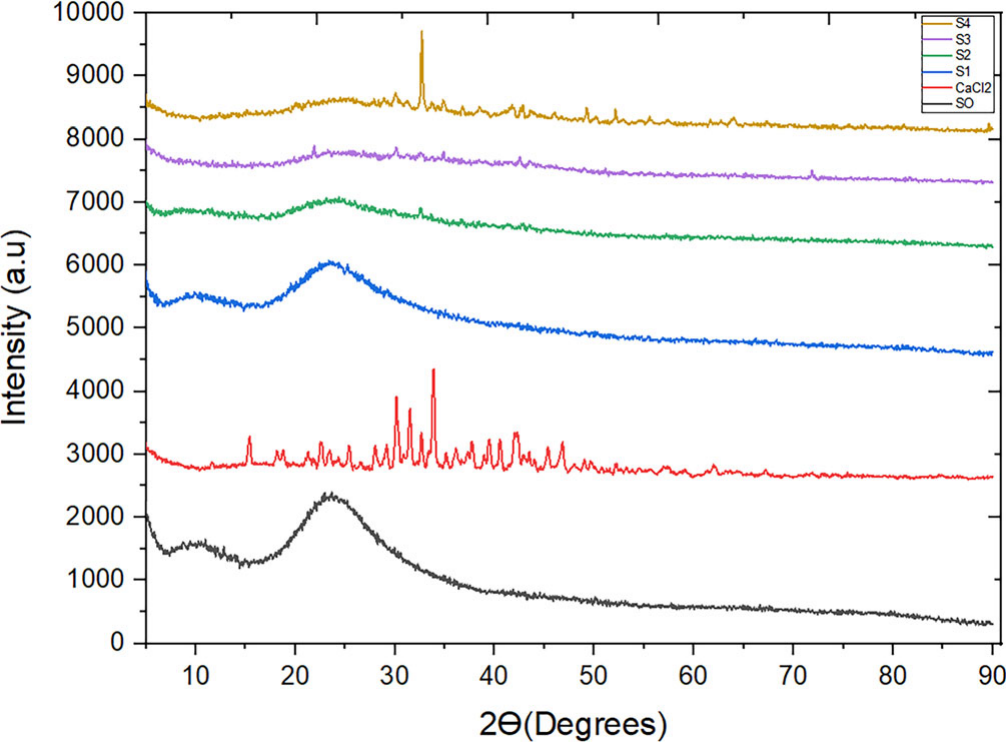
XRD curves of fabricated samples.

The pristine and composite samples were analyzed using the x‐ray diffraction technique, as depicted in Figure 3, given below. As per the XRD pattern, a typical broad peak of S0 was observed between θ = 20° and 30°, characteristic of an amorphous structure, attributed possibly to the small crystallite size and incomplete structure in the case of S0. No other impurity peaks were detected. No other peak for impurity was observed. In contrast, the XRD pattern for CaCl_2_ exhibits multiple distinct peaks at various angles, including 15°, 20.6°, 28.52°, 32.48°, 36.52°, 41.4°, 44°, 46.1°, 51.96°, and 63.85°, which are consistent with the crystalline structure of CaCl_2_ as reported in the literature (Dana, 2022; Moreira et al., 2018). These peaks serve as a reference for identifying the presence and concentration of CaCl_2_ in the composite samples.

When examining the composite curves, it becomes evident that as the concentration of CaCl_2_ salt increases, sharper peaks emerge. Initially, at 1 wt.% CaCl_2_, the peak intensities are relatively low due to the limited presence of the metal salt. However, at 2 wt.% and 3 wt.% CaCl_2_, observable peaks with somewhat reduced intensity signify the presence of the metal salt. Finally, at 4 wt.% CaCl_2_, the peaks become more pronounced and well‐defined, indicating a higher crystalline order of CaCl_2_ within the composite matrix (Dana, 2022).

### Fourier transform infrared spectroscopy (FT‐IR)

**FIGURE 4:**
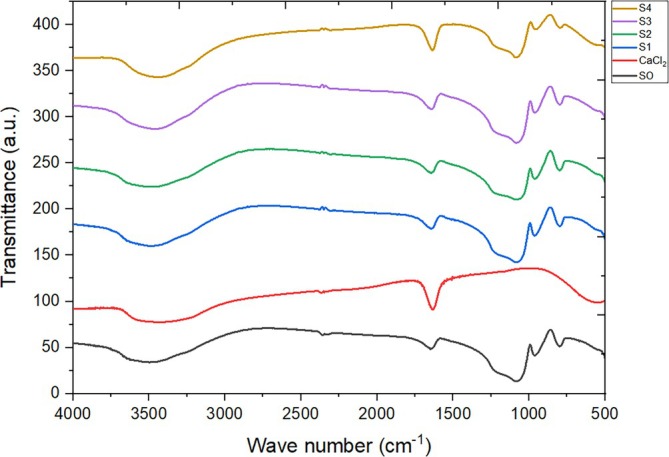
FT‐IR spectrum of fabricated adsorbents.

The qualitative analysis of pure and composite samples was conducted using the FT‐IR technique. According to the spectrum of pure silica gel as depicted in Figure 4, the peak at ~808 cm^−1^ reflects Si‐O bending vibration, whereas another peak at ~962 cm^−1^ highlights the bending vibration of (Si–O–(H⋯H_2_O). Furthermore, the asymmetric stretching vibration of the Si‐O‐Si band can be seen at ~1080 cm^−1^, a peak at ~1651 cm^−1^ shows the H–O–H bending vibration of adsorbed molecular water, and finally, a peak at ~3480 cm^−1^ reflects the Si‐OH stretching vibration, hydrogen‐bonded (Rahman et al., 2009).

The spectrum of pure salt CaCl_2_ highlighting the peaks at ~3498 cm^−1^ indicates the O‐H stretch, whereas a very small peak at ~2368 cm^−1^ reflects the O‐H stretch. The peak at ~1632 cm^−1^ is an indication of H‐O‐H bending vibrations, confirming the presence of water in CaCl_2_. This peak is more prominent in pure salt as compared to pure silica, due to the greater interaction of water with chloride ions (Araujo et al., 2021). Similar trends have been identified in the spectra of composites. All the peaks mentioned earlier are observable in the S0, S1, S2, and S3 samples. However, the intensity of the peak at approximately ~1640 cm^−1^ has increased with higher salt concentrations, indicating a greater presence of H‐O‐H bonding. These findings align with the XRD results.

### Porous structure and salt contents analysis

The porous structures and salt content ratio inside the pores of the samples were evaluated using nitrogen sorption tests at −196°C. The surface area of pristine silica is higher as compared to impregnated samples. According to Table 1. the surface area and pore volume of silica are 811 m^2^/g and 0.495 cm^3^/g, respectively, whereas as salt concentration increases from 1 wt.% to 4 wt.%, a decrease in surface area and pore volume is observed. As surface area decreased from 811 m^2^/g to 437 m^2^/g and pore volume varied from 0.495 cm^3^/g to 0.333 cm^3^/g.

**TABLE 1:** BET specific surface area (S_BET_), pore volume, micro and mesopore size, and the salt ratio in the composite.

Sample	^a^S_BET_ (m^2^/g)	Pore volume (cm^3^/g)	Salt ratio (%)	^b^Micropore volume (cm^3^/g)/ratio	^b^Mesopore volume (cm^3^/g)/ratio
S0	811	0.495	‐‐‐‐	0.211(42.6%)	0.284 (57.3%)
S1	702	0.448	9.5%	0.170 (37.9%)	0.278 (62.1%)
S2	688	0.446	9.9%	0.175 (39.3%)	0.271 (60.7%)
S3	556	0.418	16%	0.088 (21.1%)	0.330 (78.9%)
S4	437	0.333	32%	0.073 (22.8%)	0.260 (77.1%)

^a^ Surface area was calculated by using the BET method.

^b^ Pore volume, Micropore volume and Meso pore volume calculated by DFT method.

**FIGURE 5:**
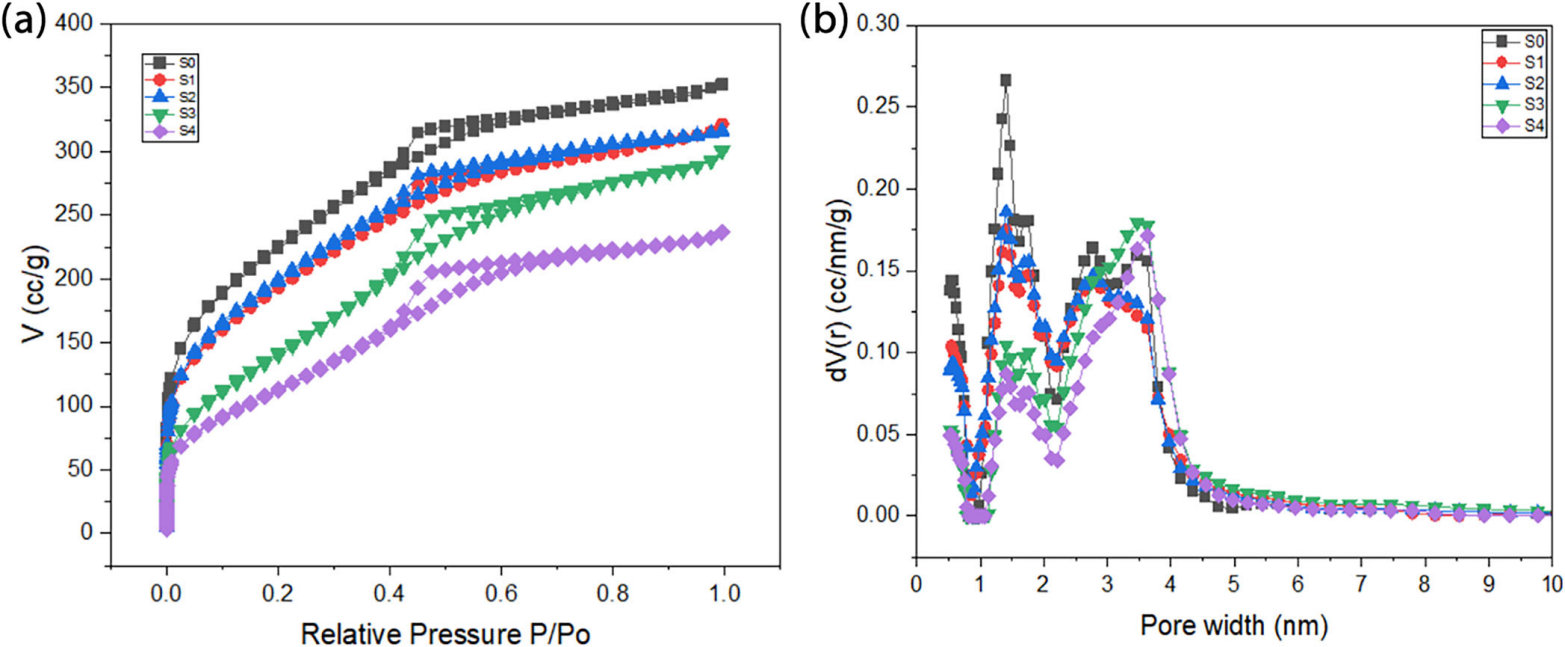
(a) N_2_ adsorption/desorption isotherms (b) pore size distribution (PSD) calculated from the N_2_ isotherms at −196°C by the DFT method.

The impact of salt impregnation in silica matrix can also be best understood by calculating the change in pore volume of matrix and composites. As per Figure 5a, the adsorption–desorption loop was observed at P/P_o_ > 0.4, which confirmed that this is a type IV isotherm in the IUPAC classification (Martín et al., 2016). This signifies the presence of mesoporous structures (0.284 cm^3^/g and 57.3%) and less microporous structures (0.211 cm^3^/g and 42.6%) in pure silica.

By evaluating the pore volumes and their ratios, it was also revealed that the same inclination of ammonium ion adsorption was observed in impregnated samples. However, with increasing the salt concentration, a reduction in micropore volume was observed. Specifically, for 1 wt.% and 2 wt.% of salt, the reduction is not very significant, but for 3 wt.% and 4 wt.%, it is high (21.1%, 22.8%). In contrast, the mesopore volume for composites has shown an increasing trend as compared to pure silica. Probably the reason is that initially, salt adsorbed more in micropores as compared to mesopores. Figure 5b confirms this claim as per DFT analysis; the reduction in the volume of micropores can be seen. These results verify that salt was successfully impregnated inside the pores of silica and also highlight the importance of salt loading in pores, which eventually enhances the mass transfer in ammonium ion adsorption.

### Ammonium ion adsorption analysis

The ammonium ion adsorption analysis was performed at room temperature by mixing 0.5 g of the adsorbent with 50 ml of the stock solution. The solutions were agitated using a shaking rotor for 60 minutes, during which equilibrium was achieved. To ensure complete adsorption and confirm the stability of the equilibrium concentration, the shaking was continued for an additional 30 minutes, for a total of 90 minutes. The equilibrium adsorption concentration was measured at the 90‐minute mark to ensure no significant changes occurred after 60 minutes, confirming that equilibrium was fully stabilized.

**FIGURE 6:**
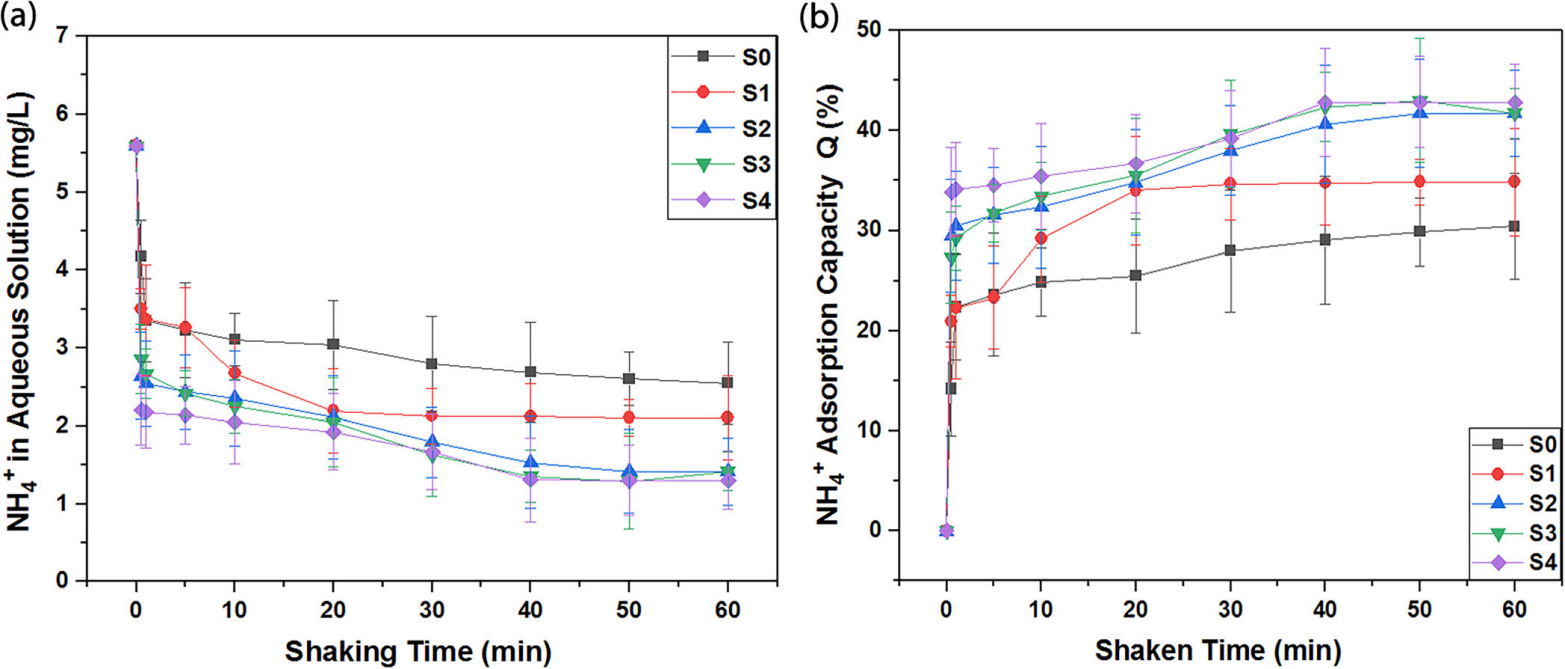
(a) Ammonium ion reduction curves in aqueous solution. (b) Ammonium ion adsorption capacity curves of pure and composites at various shaking times.

According to the results in Figure 6a,b, the adsorption of ammonium ions is directly proportional to the metal salt concentration. The figure depicts the presence of ammonium ions in solution after shaking at different times. The sudden decrease was observed during the first 10 minutes of the shaking. However, from 10 to 40 minutes, a similar trend was observed, but not that steep. From 40 to 60 minutes, the adsorption of all samples nearly stagnates. According to Figure 6, the adsorption percentage of adsorbents increased with an increment in metal salt concentration. However, initially, little difference can be observed for S0 and S1 (validated by BET analysis), but as the shaking time increases, it results in the adsorption of ammonium ions. This shaking time enhanced the contact time of CaCl_2_ salt and ammonium ions on the surface, particularly in the micro‐ and mesopores. Other than this, the hydroxyl (OH^−^) groups at the surface of silica also adsorb the ammonium ion. The adsorption of the NH_4_^+^ ion with the Cl^−^ ion is depicted in Figure 6, as the concentration of the ammonium ion decreases with shaking time (Zhou et al., 2021). This is in coordination with the BET, SEM, and XRD results of samples (Muscarella et al., 2021). Furthermore, the error bars in Figure 6a,b represent the standard deviation from three replicated experiments, confirming the consistency and reliability of the results.

### Adsorption kinetics

According to the results, the ammonium adsorption on the adsorbent follows the following main points:

- The diffusion of an ion on the external surface of the adsorbent from the bulk solution is called external mass transfer.
- Adsorb on silica and metal salt surfaces.
- Possibly adsorb at various internal surfaces.

To evaluate these points, it is imperative to explore the adsorption kinetics by considering the adsorption uptake rate and residence time and by discussing pseudo‐first‐order and pseudo‐second‐order reaction mechanisms (Ho & McKay, 1998).

Equation (3) is the pseudo‐first‐order equation.

(3) $$\log\left(q_{e}-q_{t}\right)=\log q_{e}-k_{1}t$$

Whereas the pseudo‐second‐order equation can be written as:

(4) $$\frac{dq_{t}}{\mathrm{dt}}=k_{2}\;{\left(q_{e}-q_{t}\right)}^{2}$$

By integrating this equation with the limits t = 0 to t and q_t_ = 0 to q_t_, the equation takes the following expression:

(5) $$\frac{t}{q_{t}}=\frac{1}{k_{2}{q_{e}}^{2}}+\frac{t}{q_{e}}$$

Where t is the time (s) of adsorption and q_e_ and q_t_ (mg/g) are the amounts of ammonium ions adsorbed in the membrane at equilibrium and at any time. On the other hand, the equilibrium rate constants of the first and second‐order pseudo equations are k_1_ (g/mg.s) and k_2_ (s^−1^) (Fan et al., 2003; Farrukh et al., 2017).

**FIGURE 7:**
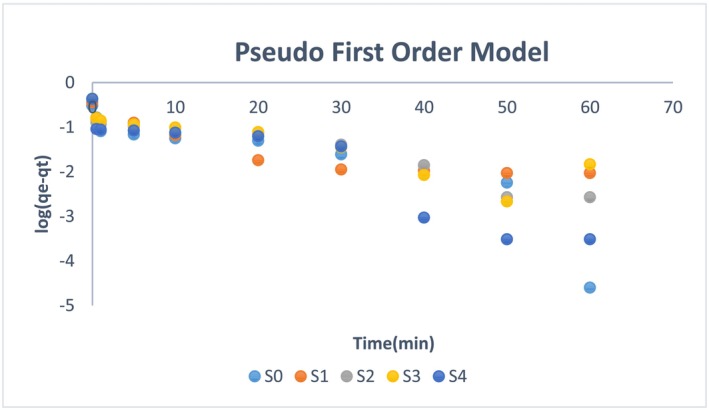
Pseudo first order model analysis.

The pseudo‐first‐order is applied to the experimental values, and the rate constant k_1_ is calculated by measuring the slope of time versus the log (q_e_ ‐ q_t_) plot (Hafeez et al., 2015). The calculated data is represented in Table 2. As per Figure 7 and the tabulated data, the ammonium ion adsorption does not follow the pseudo‐first‐order model. The correlation factor R^2^ for adsorbents ranged from 0.48 to 0.76.

**TABLE 2:** Pseudo‐order and second‐order rate constants.

Samples	Pseudo‐first‐order rate constants			Pseudo‐second‐order rate constants		
	k_1_ (s^−1^)	Rate equation	R^2^	k_2_ (g/mg.s)	Rate equation	R^2^
S0	0.0453	Log (q_e_‐q_t_) = −0.0453 t‐0.6638	0.76	1.93	t/q_t_ = 3.26 t + 5.614	0.997
S1	0.0035	Log (q_e_‐q_t_) = −0.0035 t + 0.1655	0.50	2.1	t/q_t_ = 2.79 t + 3.6178	0.999
S2	0.0317	Log (q_e_‐q_t_) = −0.0317 t − 0.6892	0.70	1.27	t/q_t_ = 2.355 t + 4.4639	0.995
S3	0.004	Log (q_e_‐q_t_) = −0.004 t + 0.1944	0.48	1.32	t/q_t_ = 2.313 t + 4.0311	0.995
S4	0.048	Log (q_e_‐q_t_) = −0.0489 t − 0.6792	0.80	1.60	t/q_t_ = 2.298 t + 3.3891	0.996

**FIGURE 8:**
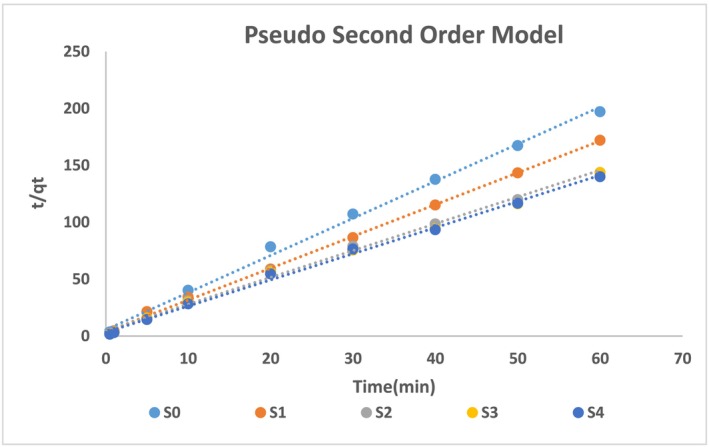
Pseudo second order model analysis.

However, by applying the pseudo‐second‐order model, the results indicated that the experimental data follow this model. As depicted in Figure 8 below, the t/q_t_ versus time plot for pure silica and silica/CaCl_2_ composite samples shows the linear curves. This curve has an intercept of 1/k_2_q_e_^2^ and a slope of 1/q_e_. The correlation factor R^2^ values fall between 0.995 and 0.996. The validation of experimental results supports the time‐transient ammonium ion profile of adsorbents.

The values of rate constants k_1_ and k_2_ have an effect on adsorption kinetics. The larger the value of k_1_, the quicker the mass transfer will be. However, the larger the value of k_2_, the slower the adsorption rate. As all the adsorbents have followed the second‐order pseudo‐order model, the value of k_1_ has no regular trend. S0 and S1 show higher k_1_ values, while S2, S3, and S4 are not showing a particular trend. On the other hand, the k_2_ value of the S1 sample is higher, indicating a slower mass transfer rate than other samples. The reason might be due to a lower amount of metal salt resulting in adsorption on metal salt and the internal surfaces of silica. However, other samples have a higher amount of salt dominating the adsorption, resulting in a fast mass transfer rate (Atamanova et al., 2022). These models highlight the rate of mass transfer in comparison and confirm the presence of physicochemical interactions in ammonium ion adsorption. However, the steps involved in the mechanism cannot be identified only by the pseudo‐order models (Li, Liao, & Zhou, 2017; Shang et al., 2018).

The adsorption kinetics need to be further evaluated by the intra‐particle diffusion model on experimental data to evaluate the steps involved. Adsorbate will diffuse from the bulk media to the adsorbent's surface, which is necessary for the adsorption process to occur. This process will involve diffusion and solubility inside the active spaces and functional groups. The model stated by (Ho & McKay, 1998) and (Ho et al., 2000) is given below.

(6) qt=kt12+C

**FIGURE 9:**
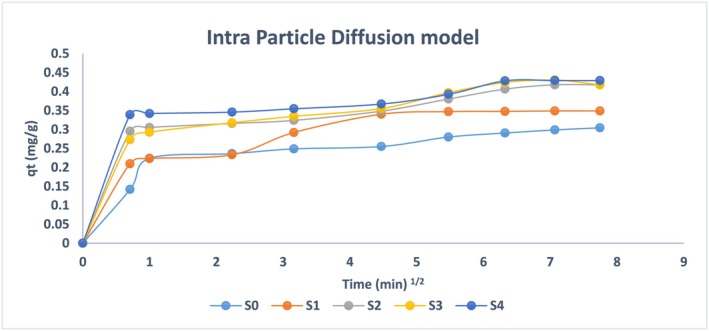
Intra particle diffusion model analysis.

In this equation, q_t_ (mg/g) is the amount of ammonium ions adsorbed at time t (s), and k is the intra‐particle rate constant (mg/s). The curves of all samples depict two different regions, explaining two different steps involved in adsorption as shown in Figure 8. The linear section ranges from 0 to 1 s, where 1/2 minute is reflecting sorption on sample surfaces, and from 5 to 60 s, where 1/2 minute is a straight line depicting the domination of intra‐particle diffusion not on the surface but inside the micro and mesopores. This may suggest that initial adsorption is predominantly diffusion on the adsorbent surface (Largitte & Pasquier, 2016). The second step involves adsorption via adsorbents impregnated inside the pores of the mesoporous matrix. The mechanism behind ammonium ion adsorption is based on enhanced penetration of ammonium ions inside the pores, which get adsorbed on impregnated CaCl_2_ salt. This is confirmed by the intra‐particle diffusion model as represented in Figure 9.

**FIGURE 10:**
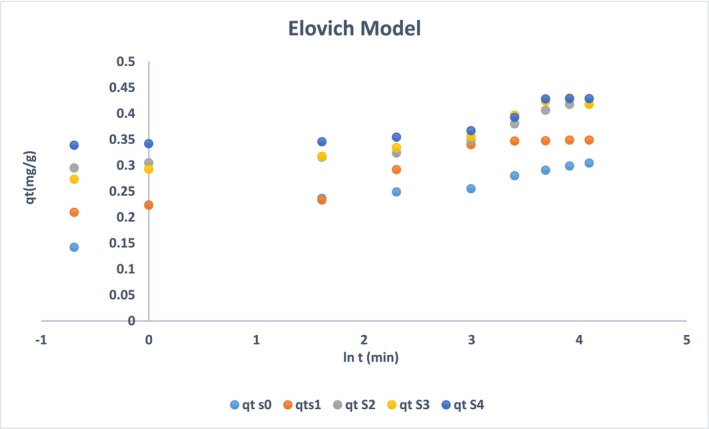
Elovich model analysis.

Other than the pseudo‐order and intra‐particle diffusion models, the Elovich model is another imperative model to analyze the adsorption of ammonium ions on adsorbents as represented in Figure 10 and Table 3 (Largitte & Pasquier, 2016; Zahedifar et al., 2010). It is stated as:

(7) $$\frac{\mathrm{dq}}{\mathrm{dt}}=\alpha \exp-\mathrm{\beta q}$$

**TABLE 3:** Elovchi model constants.

Samples	Elovich model			
	α (mg/g. s)	β (g/mg)	Rate equation	R^2^
S0	26.4	36.49	q_t_ = 0.0274 t + 0.1884	0.88
S1	23.8	29.94	q_t_ = 0.0334 t + 0.2197	0.91
S2	155	28.46	q_t_ = 0.035 t + 0.2949	0.89
S3	167	26.67	q_t_ = 0.0374 t + 0.3150	0.90
S4	173	25.06	q_t_ = 0.0399 t + 0.3343	0.87

Where q is the amount adsorbed (mg/g), α and β are constants. Assuming q = 0 and t = 0, the equation takes the form of the following equation:

(8) $$q=\left(\frac{1}{\beta }\right)\ln\left(1+\mathrm{\alpha \beta t}\right)$$

Considering αβt≫>1 the simplified equation, it can be written as below:

(9) $$\mathrm{qt}=\left(\frac{1}{\beta }\right)\ln\;\left(\alpha \beta \right)+\left(\frac{1}{\beta }\right)\ln t$$

The Elovich model represents the impact of constants. As concentration increases, the constant α will increase, whereas β decreases (Cheung et al., 2000). The increment of α represents that the initial adsorption rate is high in sample S4, and the decrement in β depicts that S4 can accommodate more adsorbents with time without hindrance. This model can guide the nature of adsorption, which is physicochemical (Largitte & Pasquier, 2016). Initially, on the surface, the silica has a silanol (SiOH) group that might undergo partial deprotonation at a pH value of 6.19. These negatively charged SiO^−^ interact with ammonium ions. Inside the pores, the ionic interaction between Cl^−^ and NH_4_^+^ ions caused indirect interaction with Ca^2+^, enhancing the ammonium ion adsorption from water. As compared with the pseudo‐second‐order model, the value of the correlation factor R^2^ is less, which signifies that ammonium ion adsorption is physicochemical in nature (Li, Liao, & Zhou, 2017; Shang et al., 2018).

### Ammonium ion adsorption from fish aquarium water

After testing pure silica and calcium chloride samples for ammonium ion adsorption and subsequent kinetic analysis, sample S4 has been tested three times with aquarium water containing ammonium ion concentrations with a pH value of 6.19 to ensure accuracy, account for variability, and calculate the standard deviation, providing reliable and consistent results while following the same process mentioned above.

**FIGURE 11:**
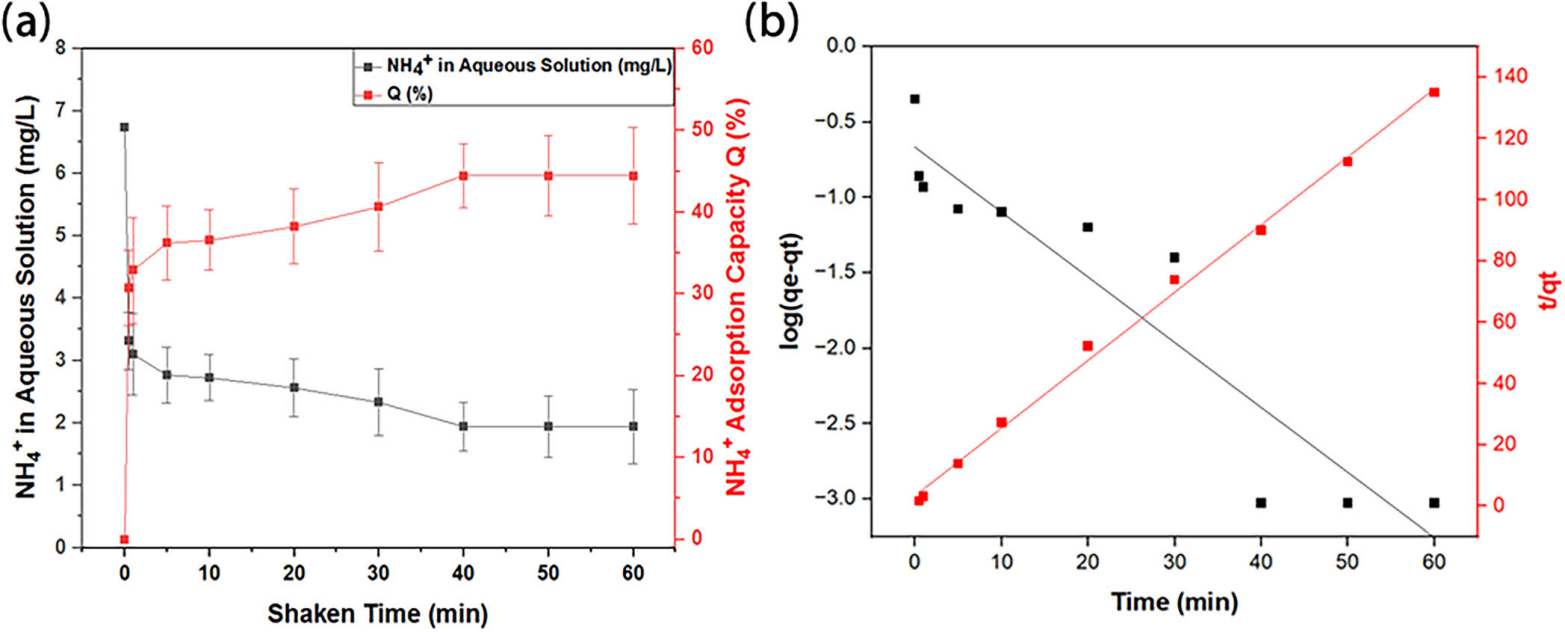
(a) Ammonium ion adsorption and reduction curves in aquarium water (b) Pseudo‐first‐order and pseudo‐second‐order analysis.

For the calibration curve, five different concentrations were analyzed, and regression analysis was applied to calculate the unknown concentrations as represented in Figure S3. The NH_4_^+^ ion adsorption can be observed in Figure 11a. The trend of the curves is nearly the same for sample S4 adsorbed from pure water. Initially, the adsorption rate is fast until 10 minutes of shaking. However, increasing shaking time increases the adsorption of ammonium ions. But the mass transfer is slow until it reaches equilibrium. As represented in Table 4, if we compare results, the adsorbed percentage of ammonium ions in sample S4 is lower in aquarium water as compared to pure water. The reason might be due to the presence of other salts in the water, which hinder the interaction between ammonium ions, mesoporous silica, and salt. Furthermore, after the treatment, the pH value increased from 6.19 to 6.74, which can be attributed to the removal of ammonium ions from the aquarium water. The increase in pH occurs because the reduction of ammonium ions (NH₄^+^) during adsorption results in a decrease in the concentration of hydrogen ions (H^+^), which leads to a higher pH. This effect has been documented in various studies, where ammonium adsorption was linked to a subsequent increase in solution pH due to the reduction of acidity as ammonium is removed (Ali et al., 2012; Leszczyński, 2021; Zhou, Wang, et al., 2024).

**TABLE 4:** Pseudo‐first‐order, pseudo‐second‐order, and Elovich models rate parameters.

Sample	Pseudo‐first ‐order rate constants			Pseudo‐second order rate constants		
	k_1_(s^−1^)	Rate equation	R^2^	k_2_ (g/mg.s)	Rate equation	R^2^
**S4**	0.0432	Log (q_e_‐q_t_) = −0.0432 t‐0.6667	0.813	1.69	t/q_t_ = 2.381 t + 3.3414	0.996

	Elovich model			
Sample	α (mg/g.s)	β (g/mg)	Rate equation	R^2^
**S4**	171	24.93	q_t_ = 0.0401 t + 0.3353	0.86

**FIGURE 12:**
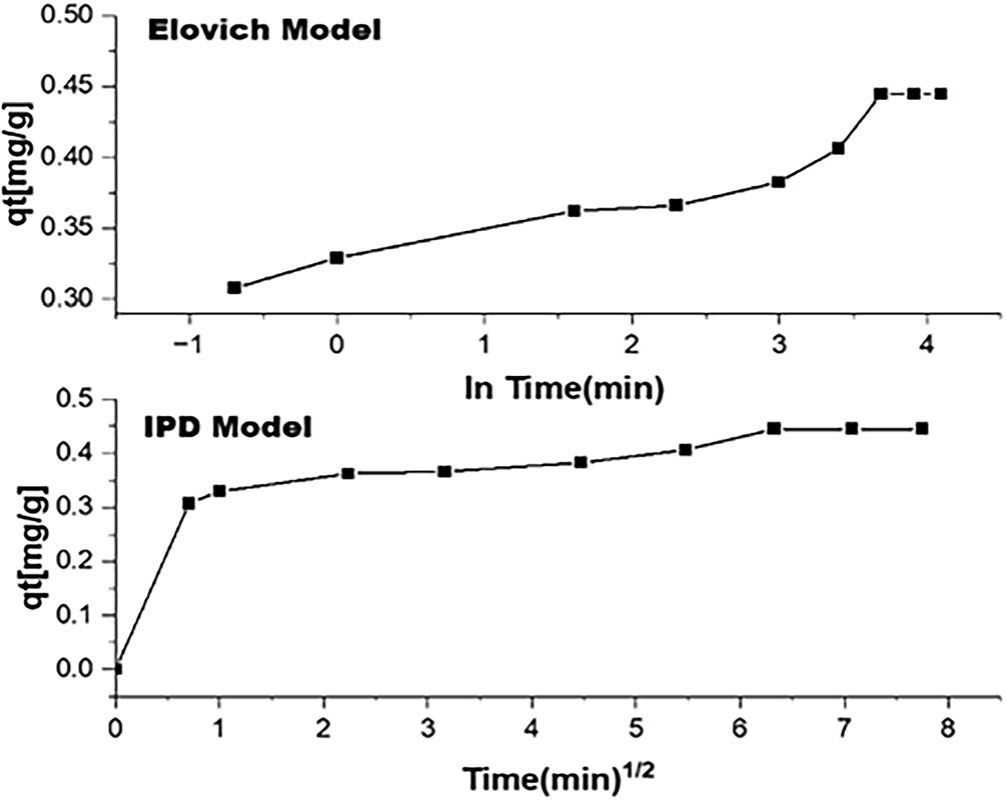
Elovich model and intraparticle diffusion model analysis.

The kinetic models, such as pseudo‐first‐order and pseudo‐second‐order, were applied to verify the experimental results, and as expected, the pseudo‐second‐order model fit well with a correlation factor value of 0.996. If we compare the rate constant K_2_ value for S4 in pure and aquarium water, an increment was observed as illustrated in Figure 11b. This highlights that the mass transfer is slow in aquarium water as compared to pure water. This validates the previously discussed point about hindrances in the interaction between ammonium ions and adsorbents. Similarly, the correlation factor value in the Elovich model is less, which confirms that physiosorption is dominated by ammonium ion adsorption as shown in Figure 12.

## CONCLUSION

In this study, pure silica and silica/calcium chloride (CaCl_2_) composite adsorbents were fabricated to investigate the adsorption of ammonium ions in water. These composites were analyzed using various techniques, including Fourier transform infrared spectroscopy (FT‐IR), scanning electron microscopy (SEM), X‐ray diffraction (XRD), thermal gravimetric analysis (TGA), and differential scanning calorimetry (DSC). Additionally, pore and salt ratio analyses were conducted using BET and DFT methods. The consistent results obtained from these analyses affirm the successful fabrication of the composite materials. Ammonium ion adsorption was specifically examined using the UV/VIS technique, with successful calibration achieved at a wavelength of 500 nm. The adsorption results are in correlation with the concentration of metal salt and the increased adsorption of ammonium ions.

Whereas the kinetic analysis has provided additional insights. According to the pseudo‐second‐order model, it was observed that the mass transfer rate for sample S1 is slower compared to S2, S3, and S4. This slow mass transfer in S1 is likely attributed to the extended time required for surface and internal interactions. Conversely, samples S2, S3, and S4 exhibited a more comparable mass transfer rate, suggesting a more efficient process due to a higher quantity of metal salt on the surface. The intramolecular model confirmed that other than surface adsorption, intramolecular adsorption is also taking part in this process, which confirms the two‐step adsorption mechanism. Whereas the Elovchi Model confirms that the adsorption of ammonium ions on pure and silica/CaCl_2_ composites is a physisorption phenomenon, which is in coordination with previous kinetic models.

Furthermore, the optimum composite sample S4 was also tested for the treatment of aquarium water, which demonstrated remarkable efficiency in removing ammonium ions. The treatment process increased the pH from 6.19 to 6.74 and successfully removed up to 43% of ammonium ions. These findings highlight the potential of silica/CaCl_2_ composites as a promising option for aquarium water purification, offering a significant advancement in maintaining a safe and healthy environment for aquatic life.

## AUTHOR CONTRIBUTIONS

**Sarah Farrukh:** Conceptualization; methodology; formal Analysis and investigation; resources; writing—original draft preparation; writing—review and editing; visualization. **Xianfeng Fan:** Resources; funding acquisition; supervision. **Zhibin Yu:** Resources; funding acquisition; writing—review and editing. **Syed Shujaat Karim:** Writing—original draft preparation; writing—review and editing; visualization. **Humais Roafi:** Writing—review and editing. Zhibin Yu: Writing—review and editing.

## CONFLICT OF INTEREST STATEMENT

The authors declared that they have no conflict of interest.

## ETHICS STATEMENT

Not applicable.

## CONSENT TO PARTICIPATE

All authors have given their consent to publish the manuscript.

## CONSENT TO PUBLISH

All authors have approved the manuscript for publication.

## Supporting information


**Figure S1** (a) Ammonium ion reduction curves in aqueous solution. (b) Ammonium ion adsorption capacity curves of pure and composites at various shaking times
**Figure S2** UV/Vis calibration curve for ammonium ion adsorption
**Figure S3** UV/Vis calibration curve for ammonium ion adsorption in aquarium water

## Data Availability

The datasets used and analyzed during the current research study will be made available on reasonable request.
