# Supplementary material for: Harnessing synergistic two‐step adsorption of silica/calcium chloride (CaCl2) hybrid composites for ammonium removal from aquarium water
Source: Water Environ Res. 2025 Jun 1;97(6):e70080. doi: 10.1002/wer.70080 (PMC12127048; doi:10.1002/wer.70080)
Supplement: Supplementary file 1 — Figure S1 (a) Ammonium ion reduction curves in aqueous solution. (b) Ammonium ion adsorption capacity curves of pure and composites at various shaking times Figure S2 UV/Vis calibration curve for ammonium ion adsorption Figure S3 UV/Vis calibration curve for ammonium ion adsorption in aquarium water [file WER-97-e70080-s001.docx]

**Explanation for Calcium Chloride (CaCl_2_) - Salt Percentage Range Selection**

The range of calcium chloride (CaCl_2_) salt percentages from 0 wt.% to 4 wt.% was chosen after conducting preliminary experiments and analyzing the structural and adsorption performance of the composite materials. As shown in the Figure 1S, higher concentrations of CaCl_2_, such as 5 wt.%, resulted in pore blockage within the silica/CaCl_2_ composite, which hindered the accessibility of ammonium ions to the adsorbent's porous structure. This reduction in pore accessibility decreased the overall ammonium adsorption efficiency of the material. Therefore, the selected range of 0 wt.% to 4 wt.% CaCl_2_ was optimized to avoid these adverse effects while ensuring that the composites maintained their structural integrity and adsorption capabilities. This selection was crucial in achieving a high-performing adsorbent suitable for ammonium removal from aqueous solutions, particularly in aquaculture applications.


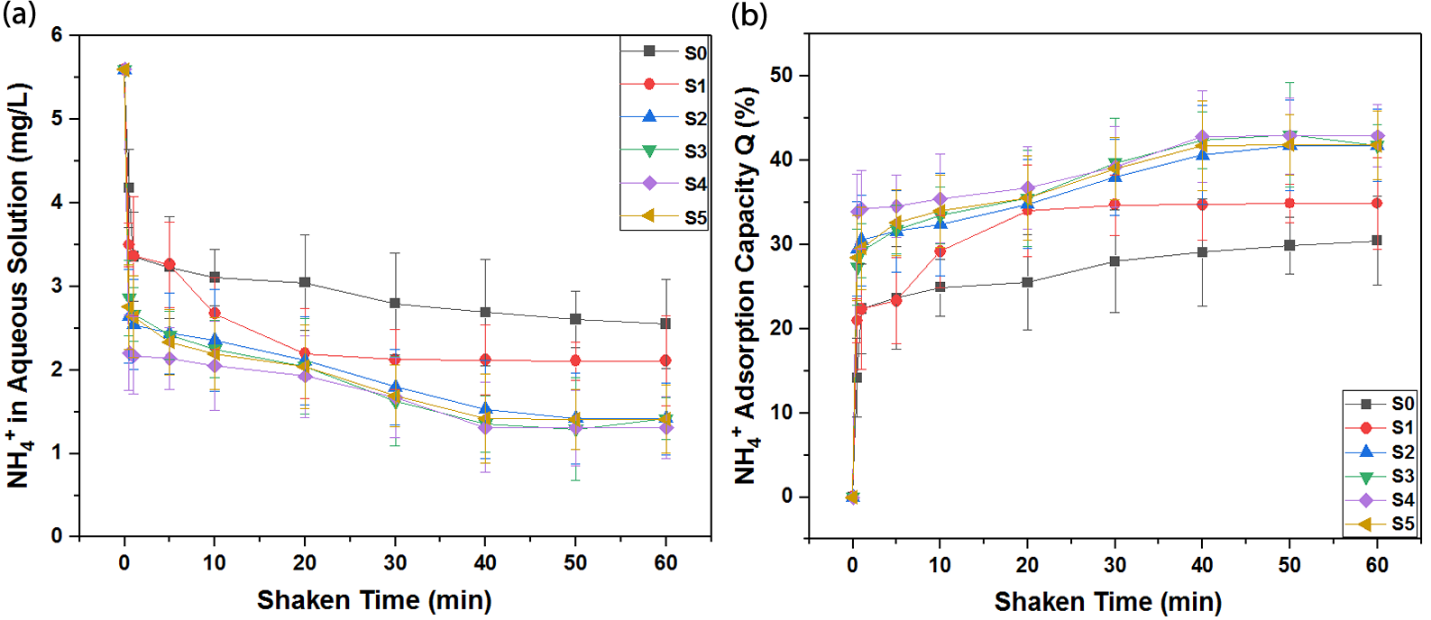


**Figure. 1S (a) Ammonium ion reduction curves in aqueous solution (b) Ammonium ion adsorption capacity curves of pure and composites at various shaking times**

**
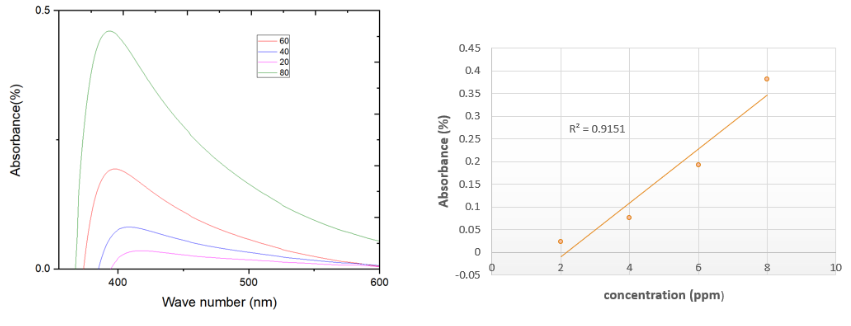
**

**Figure 2S UV/Vis Calibration curve for ammonium ion adsorption**

**Figure 3S UV/Vis Calibration curve for ammonium ion adsorption in aquarium water**
